# Polymorphism in *LEP* and *LEPR* May Modify Leptin Levels and Represent Risk Factors for Thyroid Cancer

**DOI:** 10.1155/2015/173218

**Published:** 2015-02-25

**Authors:** Marjory Alana Marcello, Antonio Ramos Calixto, Jacqueline Fatima Martins de Almeida, Mariana Bonjiorno Martins, Lucas Leite Cunha, Camila Ayume Amano Cavalari, Elba C. S. Etchebehere, Ligia Vera Montalli da Assumpção, Bruno Geloneze, Andre Lopes Carvalho, Laura Sterian Ward

**Affiliations:** ^1^Laboratory of Cancer Molecular Genetics (Gemoca), Faculty of Medical Sciences, University of Campinas (FCM-Unicamp), Rua Tessalia Vieira de Camargo 126, 13083-970 Campinas, SP, Brazil; ^2^Laboratory of Investigation on Metabolism and Diabetes (LIMED), Faculty of Medical Sciences, University of Campinas (FCM-Unicamp), Rua Carlos Chagas 420, 13083-878 Campinas, SP, Brazil; ^3^Service of Nuclear Medicine, University of Campinas, Rua Vital Brasil 251, 13083-888 Campinas, SP, Brazil; ^4^Service of Endocrinology, University of Campinas, Rua Vital Brasil 251, 13083-888 Campinas, SP, Brazil; ^5^Department of Head and Neck Surgery, Barretos Cancer Hospital, Rua Antenor Duarte Vilela 1331, 14784-400 Barretos, SP, Brazil

## Abstract

*Purpose.* To understand the role of polymorphisms in the LEP (rs7799039 and rs2167270) and LEPR (rs1137101 and rs1137100) genes in DTC susceptibility and their effect on leptin levels. *Methods.* We studied 153 patients with DTC and 234 controls through TaqMan SNP Genotyping and ELISA, comparing these data to the clinicopathological data of patients with DTC. *Results.* Patients with AA genotype of rs7799039 had higher levels of serum leptin (9.22 ± 0.98 ng/mL) than those with AG genotype (10.07 ± 0.60 ng/mL; *P* = 0.005). Individuals with AG genotype of rs2167270 also produced higher serum leptin levels (10.05 ± 0.59 ng/mL) than the subjects with GG genotype (9.52 ± 0.79 ng/mL; *P* < 0.05). A multivariate logistic regression adjusted for gender, age, and BMI showed that the AG genotype of rs7799039 was an independent risk for DTC (OR, 11.689; *P* = 0.0183; 95% CI, 1.516–90.119). Similarly, AG and GG genotypes of rs1137101 increased the susceptibility to DTC (OR, 3.747; *P* = 0.027; 95% CI, 1.161–12.092 and OR, 5.437; *P* = 0.013; 95% CI, 1.426–20.729). *Conclusions.* We demonstrated that rs7799039 and rs2167270 polymorphisms modify the serum leptin concentrations in patients with DTC. Furthermore, polymorphisms rs7799039 and rs1137101 increase the risk of DTC development, although they do not correlate with tumor aggressiveness.

## 1. Introduction

The mechanisms underlying the relationship between cancer and obesity have not been established and may vary according to the primary tumour location [1, 2]. These variations may be attributed to the metabolic and endocrine effects that obesity may exert on the metabolism, hormones, inflammation, and protein production [3], thus leading to the development and progression of cancer.

Our group has previously demonstrated that cytokines produced by adipocytes (adipokines), such as adiponectin, leptin, and resistin, are differentially expressed in malignant and benign thyroid nodules, helping to differentiate not only malignancy but also subtypes of thyroid nodules, which are difficult to identify using PAAF [4]. Of the three adipokines, leptin presents as an interesting diagnostic option for thyroid malignancy; leptin has a 100% accuracy, as well as high sensitivity and specificity. Additionally, the serum leptin levels differed in follicular lesions, which is currently the primary diagnostic challenge for pathologists, introducing a fascinating clinical perspective to the diagnosis of thyroid malignancy using a simple and robust blood test [4].

Physiological mechanisms may influence the synthesis of leptin and thus lead to variations in the amounts of leptin associated with the fat mass [5]. Notably, several cytokines, such as tumour necrosis factor alpha (TNF-*α*), increase the expression of leptin through mRNA synthesis [6]. Additionally, recent evidence has indicated that the modulation of leptin's gene expression might be related to the presence of polymorphisms in* LEP* and* LEPR* (leptin receptor) gene [7, 8]. The polymorphisms in these genes have been recently related to an increased susceptibility to several types of cancer, such as prostate, breast, gastric, and lung carcinomas [9–13]. Hence, we hypothesised that polymorphisms in* LEP* and* LEPR* could lead to an increased risk for differentiated thyroid cancer (DTC), thus establishing the link between the observed epidemiological increases in both thyroid cancer and obesity rates.

The objective of the present study was to investigate the polymorphisms in* LEP* and* LEPR* genes in patients with DTC, correlating the obtained data with previously measured leptin serum levels, DTC risk, and clinicopathological features.

## 2. Subjects and Methods

### 2.1. Patients

This study was approved by the Research Ethics Committee of the participating centres. All of the participants signed the informed consent forms. We investigated 153 patients (130 females, 23 males, 40.87 ± 13.80 years) diagnosed with DTC and 234 healthy controls (204 females, 30 males, 37.61 ± 13.39 years) with no significant differences in gender, age, and body mass index (BMI). Seventy-four of 153 patients included in this study had been previously described in another report and had serum leptin levels measured [4].

The patients were consecutively referred to the Thyroid Cancer Unit, Division of Endocrinology, Unicamp Teaching Hospital in Campinas, São Paulo, Brazil, and to the Head and Neck Department of the Barretos Cancer Hospital, Barretos, São Paulo, Brazil, between 2009 and 2011, for thyroid nodule diagnostic evaluations. They were submitted to interviews, using a structured in-person questionnaire that allowed the recollection of information, such as demographic, social, economic, and xenobiotic exposures, food intake, and previous disorders. The questionnaire has been used in several publications by our group in recent years [14–17].

To classify BMI, height was measured with the individuals standing barefoot on a stadiometer with a centimetre scale. Weight was measured using a balance with division of 100 g, with all of the individuals wearing light clothes and no shoes. BMI was calculated using the formula (weight)/(height)^2^. All of the participants were classified using the WHO recommendation for nutritional status as follows: normal weight, 18.5–24.99 kg/m^2^; overweight, ≥25–29.99 kg/m^2^; and obese, ≥30 kg/m^2^ [18]. Underweight patients (<18.49 kg/m^2^) were excluded from the study because we could not obtain underweight controls.

Anatomopathological data, including nodule size, tumour histological features and image, as well as laboratory data were retrieved from the patients' medical records. All thyroid cancer histological samples were reviewed for diagnostic confirmation by a thyroid pathologist (J. Vassallo). Chronic lymphocytic thyroiditis, investigated in the nonmalignant parenchyma of the contralateral thyroid lobe, was characterised by extensive lymphocytic infiltration with lymphoid follicles, scarring, and follicular regenerative activity in the form of numerous small follicles, frequently lined by Hurthle cells.

Thyroid cancer patients were monitored using periodic total body scans and serum TSH and thyroglobulin (Tg) measurements, according to a standard protocol based on the American Thyroid Association and Latin American Thyroid Association recommendations [19, 20], which included X-ray, ultrasonography, computed tomography scans, and other timely and necessary procedures for detecting distant metastasis for a period of 18–56 months (40.8 ± 16 months). The aggressiveness of the cancer at the time of diagnosis was ascertained using the TNM stage classification system for differentiated thyroid carcinoma [21]. The patients were also classified into low, intermediate, and high risk groups, according to the ATA guidelines [19]. The patients who presented with suspicious or high serum Tg levels (>2 ng/dL) underwent intensive imaging procedure. We defined tumours as recurrent and long-distance metastasis according to the above-mentioned parameters. The patients with thyroid cancer were classified as disease-free when they maintained serum Tg levels <2 ng/dL and exhibited no clinical or image suspicion of disease for at least 12 consecutive months after surgery. There were 80 patients (63.5%) who were free of disease and 46 patients (37.5%) with recurrence. Twenty-seven patients could not be classified into any of these two groups and were excluded from any analysis involving outcome. No patient died of the disease during our follow-up.

Healthy individuals included in the control group presented neither history of malignancy in the family nor familial history of thyroid diseases. These individuals were blood donors of the Hematology and Hemotherapy Center, located in the University of Campinas.

### 2.2. Serum Analysis

The 74 individuals investigated for serum leptin levels have undergone similar blood collection procedures, based on the previously described method [22–24]; that is, 4 mL of whole blood was collected in red plastic tubes that had no anticoagulant factors and contained a gel with intermediate density between blood cells and serum. The serum samples were then separated by centrifugation at 3000 rpm for 10 min and stored at −20°C, until analysis could be performed. We used commercial ELISA assays to quantify the circulating leptin levels (R&D Systems, Minneapolis, MN, USA).

### 2.3. Genotype Analysis

Approximately 4 mL of peripheral blood was collected into EDTA (ethylenediaminetetraacetic acid) coated tubes. Genomic DNA was extracted from leucocytes separated from whole blood, using a standard protocol, which includes leucocyte lysis, SDS treatment, phenol-chloroform extraction, and ethanol precipitation.

The DNA samples were genotyped for* LEP* (rs7799039 and rs2167270) and* LEPR* (rs1137101 and rs1137100) genes using TaqMan SNP genotyping assays (C____1328079_10, C____15966471_20, C____8722581_10, and C____518168_20, resp.) (Applied Biosystems, CA, USA) in the 7500* Real Time* PCR* System* (Applied Biosystems, CA, USA). The reactions were carried out using a 25 *μ*L total volume containing 20 ng of sample DNA, 12.5 *μ*L of TaqMan universal PCR* Master Mix*, 0,625 *μ*L of TaqMan assay, and 8.875 *μ*L of milli-Q water. The reactions were then analysed using the allelic discrimination endpoint analysis of the sequence detection software package,* Sequence Detection Software* (SDS) Version 1.3 (Applied Biosystems). These SNPs were selected because previous studies showed that they were associated with other cancers, obesity-related diseases, and leptin expression [9, 25–30].

### 2.4. Statistical Analysis

The statistical analysis was conducted using the SAS statistical software (Statistical Analysis System, version 8.1, 1999-2000, Cary, NC, USA). Associations were assessed using 2 × 2 or 2 × *n* contingency table analyses, and *χ* ^2^ or Fisher's exact test was used to examine homogeneity between cases and controls. Kruskal-Wallis and Mann-Whitney tests were used to compare the mean ages of patients and controls and to compare the mean serum leptin expression among genotypes. The odds ratio (OR) and 95% CI provided the measurements of association strength. The variables that were significantly associated with DTC using univariate analysis were analysed using a multiple logistic regression model for evaluating the effect of all genotypes and clinical risk factors. To analyse the primary factors related to the disease-free interval, Cox regression analysis was performed. All of the tests were conducted at the *P* < 0.05 significance level.

## 3. Results

The majority (82%) of the patients were females, aged 14 years to 76 years (41.02 ± 15.54 years) and 87% had an anticipated diagnosis of papillary thyroid carcinoma (PTC). Multifocality was observed in 55% of the patients and 59% presented invasion of the capsule. Stage I (40%) and stage II (35%) were more frequent, although at the reference hospitals involved in this study there were a large proportion of stage III (10%) and stage IV (15%) cases. In fact, 37 patients presented metastasis at the time of diagnosis. Moreover, concurrent thyroiditis was diagnosed in 48% of the patients. The majority (63.5%) of the patients evolved free of disease after a 9-year follow-up period (101.5 ± 57.6 months). However, 46 patients (36.5%) presented recurrence or metastasis during the follow-up.

### 3.1. Serum Analysis

As we have previously described, leptin presented outstanding accuracy in the diagnosis of thyroid nodule malignancy [4]. All of the parameters considered for a diagnostic test were noticeable. For a cut-off point of 7.24 ng/mL, the serum levels of leptin differentiated all of the malignant lesions from the benign lesions, with sensitivity, specificity, and positive predictive value and negative predictive value of 100% (*P* = 1 × 10^−6^). Additionally, leptin serum levels differentiated the follicular variant of papillary thyroid cancer (FVPTC) from the follicular adenomas (FA) (*P* < 0.001) and from goiters (*P* < 0.001). They also distinguished FA from the follicular carcinomas (FTCs) (*P* < 0.001) and from the classic PTC (CPTC) (*P* < 0.001) [4]. These results are shown in Table 1. Although leptin was differentially expressed among thyroid lesions, it did not correlate with any clinicopathological feature (Table 2).

### 3.2. Genotyping Analysis

A multivariate logistic regression adjusted for gender, age, smoking, and BMI showed that the AG genotype of* LEP* rs7799039 was an independent risk for DTC (OR, 11.689; *P* = 0.0183; 95% CI, 1.516–90.119). Similarly, AG and GG genotypes of* LEPR* rs1137101 represented independent risks for DTC (OR, 3.747; *P* = 0.027; 95% CI, 1.161 to 12.092, and OR, 5.437; *P* = 0.013; 95% CI, 1.426 to 20.729, resp.).

The AA genotype of* LEPR* rs1137100 was more frequent among patients with FTC (93.8%) than among patients with PTC (60.2%; chi-square = 8.219, *P* = 0.016). There was no association between the genotypes and the presence of features that could be related to aggressiveness, such as multifocality, invasion of the capsule, and concomitant thyroiditis. However, the GG genotype of rs2167270 of* LEP* gene was more frequent among the patients diagnosed with the less advanced stage (I and II, 46.2%) than among the patients diagnosed with the more advanced stage (III and IV, 22.7%, chi-square = 6,311; *P* = 0.043), as shown in Table 2.

The survival analysis showed no association between the disease-free interval and gender, ethnicity, smoking, multifocality, invasion of capsule, and thyroiditis. None of the genotypes investigated was associated with the disease-free period.

### 3.3. Leptin Concentrations versus Genotypic Profile

*LEPR* polymorphisms did not correlate with the serum concentrations of leptin as shown in Table 3. In contrast, we observed a good correlation between* LEP* gene genotype and serum leptin levels. The patients who presented the AA genotype of rs7799039 in* LEP* gene had lower serum levels of leptin (9.22 ± 0.98 ng/mL) than those with the AG genotype (10.07 ± 0.60 ng/mL; *P* = 0.005). The individuals with the AG genotype of SNP rs2167270 also produced higher serum leptin levels (10.05 ± 0.59 ng/mL) than the subjects with the GG genotype (9.52 ± 0.79 ng/mL; *P* < 0.05).

## 4. Discussion

It is recognised that leptin is a component of a series of important thyroid cell processes, including the regulation of TSH expression [31] and the development of autoimmunity [32, 33], and that serum leptin levels may be related to the presence of insulin resistance in hypothyroidism [34] and to other diseases that may be associated with thyroid disorders, such as obesity and insulin resistance. However, the present study is the first to investigate the occurrence of genetic changes in* LEP* and* LEPR* genes in DTC, which might be a likely link between obesity and thyroid cancer.

To the best of our knowledge, we demonstrate for the first time that polymorphisms in* LEP* (rs7799039) and* LEPR* (rs1137101) may increase the risk of DTC development.

The SNP rs7799039 in* LEP* gene has been associated with an increase in BMI, overweight, and even with variations in serum leptin concentration [27, 35, 36]. A meta-analysis that included 5 articles related to this SNP and colorectal carcinoma risk and 3 studies related to prostate cancer susceptibility concluded that this alteration in* LEP* may increase the risk for prostate cancer but not for colorectal carcinoma [9]. Additionally, several authors have attempted unsuccessfully to establish a relationship between this polymorphism and breast cancer [37].

We demonstrated that serum leptin concentration varies according to specific inherited genotypes. Hoffsted et al. [38] showed that individuals with the AA genotype of rs7799039 had higher serum leptin concentrations than the AG or GG genotypes carriers. Our data confirmed significant differences of this cytokine depending on the genotype, although with more discrete variations. Because this SNP is located in the promoter region, this polymorphism may affect gene expression at a transcriptional level, leading to more or less leptin production. These findings may also explain the fact that there was no correlation between serum leptin and BMI in our population, allowing us to hypothesise that changes in this gene may be even more important than the effects of obesity in the secretion of this hormone.

Regarding SNP rs1137101, which we demonstrated to be related to an increased risk for DTC, reports in the literature have shown that variants of* LEPR* are related to the following: increased BMI, insulin resistance, and correlation with HOMA-IR (an index used for the assessment of insulin resistance), metabolic syndrome, increased fat mass and adipocytes, increased waist circumference, obesity, development of type 2 diabetes mellitus (T2DM), and serum concentrations of circulating leptin [39–52]. This polymorphism has been associated with breast, prostate, and lung cancers and polycystic ovary syndrome [53–59].

We described the relationship of rs2167270 of* LEP* with different serum levels of leptin. Friedlander et al. [60] correlated LEP rs2167270 genotypes with increased body mass and waist circumference in women, and Jiang et al. [27] reported that this polymorphism is associated with increased BMI. The AA genotype of rs2167270 was associated with a lower risk of colorectal cancer [25]. Although the variations in* LEP* may alter the amount of leptin produced, no relationship was found between rs2167270 and increased risk for DTC.

In conclusion, we demonstrated that rs7799039 and rs2167270 polymorphisms of* LEP* modified the serum concentrations of leptin in patients with DTC. Furthermore, the polymorphisms rs7799039 in* LEP* and rs1137101 in* LEPR* increased the risk for developing DTC, although they did not appear to correlate with tumour aggressiveness.

## Figures and Tables

**Table 1:** Comparisons of different histopathological types according to their leptin serum expression.

Analysed groups	Leptin	*P*
Malignant versus benign	9.89 ± 0.63 versus 1.92 ± 0.69	**<0.001**
CPTC versus goiter	9.69 ± 0.89 versus 1.88 ± 0.73	**<0.001**
CPTC versus FA	9.69 ± 0.89 versus 2.11 ± 0.47	**<0.01**
CPTC versus FC	9.69 ± 0.89 versus 9.49 ± 0.50	N.S.
FVPTC versus CPTC	10.02 ± 0.57 versus 9.69 ± 0.89	N.S.
FVPTC versus goiter	10.02 ± 0.57 versus 1.88 ± 0.73	**<0.001**
FVPTC versus FA	10.02 ± 0.57 versus 2.11 ± 0.47	**<0.001**
FVPTC versus FC	10.02 ± 0.57 versus 9.49 ± 0.50	N.S.
FC versus FA	9.49 ± 0.50 versus 2.11 ± 0.47	**<0.001**
FC versus goiter	9.49 ± 0.50 versus 1.88 ± 0.73	**<0.01**
FA versus goiter	2.11 ± 0.47 versus 1.88 ± 0.73	N.S.

CPTC: classic type PTC; FVPTC: follicular variant PTC; FC: follicular carcinoma; AF: follicular adenoma; N.S.: not statistically significant.

**Table 2:** Leptin serum concentrations and *LEP* and *LEPR* SNPs genotypes compared to clinicopathological features.

Clinical pathological features	Leptin serum concentration (ng/mL)	*P*	*LEP * rs7799039 (*N*)			*P*	*LEP * rs2167270 (*N*)			*P*	*LEPR * rs1137101 (*N*)			*P*	*LEPR * rs1137100 (*N*)			*P*
			AA	AG	GG		AA	AG	GG		AA	AG	GG		AA	AG	GG	
Age at time of diagnosis																		
≤45	9.81 ± 0.58	N.S.	11	39	40	N.S.	8	48	39	N.S.	24	47	24	N.S.	65	20	3	N.S.
>45	10.07 ± 0.80		10	33	20		11	32	24		19	30	18		33	25	6	
Gender																		
Male	9.88 ± 0.60	N.S.	17	57	44	N.S.	11	63	46	N.S.	29	56	35	N.S.	69	37	6	N.S.
Female	9.90 ± 0.80		4	8	10		2	7	13		7	10	5		18	3	1	
Smoking habit																		
Smokers	9.98 ± 0.76	N.S.	7	20	12	N.S.	2	18	20	N.S.	12	17	11	N.S.	26	10	2	N.S.
Never smoked	9.82 ± 0.58		13	45	40		11	50	38		23	48	28		59	30	4	
Tumour size																		
<2 cm	9.99 ± 0.58	N.S.	14	31	29	N.S.	9	34	31	N.S.	21	35	18	N.S.	44	21	5	N.S.
2–4 cm	9.73 ± 0.77		8	33	25		6	38	23		13	38	16		42	16	2	
>4 cm	9.95 ± 0.38		1	15	14		3	17	11		9	13	9		16	9	0	
Extrathyroidal invasion																		
Yes	9.94 ± 0.70	N.S.	8	30	15	N.S.	5	31	19	N.S.	16	22	17	N.S.	32	17	1	N.S.
No	9.87 ± 0.62		16	38	17		46	20	3		8	29	34		7	34	30	
Capsule																		
Yes	9.80 ± 0.76	N.S.	8	17	14	N.S.	5	17	17	N.S.	11	17	11	N.S.	24	12	1	N.S.
No	9.96 ± 0.57		1	17	10		1	20	7		5	17	6		19	6	1	
Multifocality																		
Yes	9.82 ± 0.67	N.S.	15	27	29	N.S.	9	34	29	N.S.	19	37	16	N.S.	44	19	4	N.S.
No	9.88 ± 0.65		7	34	22		5	31	28		16	27	21		42	18	1	
Metastasis at time of diagnosis																		
Present	9.86 ± 0.83	N.S.	3	16	17	N.S.	2	23	13	N.S.	7	18	13	N.S.	24	9	3	N.S.
Absent	9.89 ± 0.58		16	48	32		11	45	40		30	45	21		60	31	1	
Stage																		
I and II	9.60 ± 0.74	N.S.	6	32	29	N.S.	3	34	32	**0.043**	19	29	21	N.S.	42	22	2	N.S.
III and IV	10.17 ± 0.75		2	16	5		4	14	5		6	13	4		13	9	0	
Thyroiditis																		
Present	9.96 ± 0.64	N.S.	9	23	12	N.S.	4	21	20	N.S.	9	25	11	N.S.	28	11	2	N.S.
Absent	9.84 ± 0.39		5	24	24		7	25	22		20	21	13		34	19	0	
Outcome																		
Disease-free	9.88 ± 0.61	N.S.	14	43	28	N.S.	8	40	37	N.S.	27	40	18	N.S.	49	30	2	N.S.
Recurrence	9.89 ± 0.82		4	21	21		5	28	15		9	23	16		35	9	2	

N.S.: not statistically significant.

**Table 3:** Mean and median leptin serum expressions according to the genotypes of the studied SNPs.

Gene-rs (*N*)	Mean expression (ng/mL)	Standard deviation	Median (ng/mL)	*P*
*LEP *rs7799039 (78)				**0.012** ^*^
AA (12)	9.22	0.98	9.37	**<0.05** ^ #^
AG (36)	10.07	0.60	10.07	>0.05^$^
GG (30)	9.75	0.56	9.84	>0.05^$^
*LEP *rs2167270 (78)				**0.018** ^*^
AA (8)	9.89	0.49	9.80	>0.05^#^
AG (38)	10.05	0.59	10.00	**<0.05** ^ &^
GG (32)	9.52	0.79	9.58	>0.05^$^
*LEPR *rs1137101 (78)				N.S.
AA (20)	9.75	0.59	9.83	
AG (40)	9.89	0.76	9.85	
GG (18)	9.74	0.74	9.96	
*LEPR *rs1137100 (73)				N.S.
AA (48)	9.88	0.76	9.96	
AG (20)	9.77	0.72	9.84	
GG (5)	9.76	0.42	9.86	

^*^AA × AG × GG; ^#^AA × AG; ^&^AG × GG; ^$^AA × GG; N.S.: nonsignificant.

## References

[1] Cheng S. P., Chi C. W., Tzen C. Y. (2010). Clinicopathologic significance of leptin and leptin receptor expressions in papillary thyroid carcinoma. *Surgery*.

[2] Wolin K. Y., Carson K., Colditz G. A. (2010). Obesity and cancer. *The Oncologist*.

[3] Osorio-Costa F., Rocha G. Z., Dias M. M., Carvalheira J. B. (2009). Epidemiological and molecular mechanisms aspects linking obesity and cancer. *Arquivos Brasileiros de Endocrinologia & Metabologia*.

[4] Marcello M. A., Cunha L. L., Batista F. A., Ward L. S. (2014). Obesity and thyroid cancer. *Endocrine-Related Cancer*.

[5] Negrão A. B., Licinio J. (2000). Leptina: o diálogo entre adipócitos e neurônios. *Arquivos Brasileiros de Endocrinologia & Metabologia*.

[6] Finck B. N., Johnson R. W. (2000). Tumor necrosis factor-alpha regulates secretion of the adipocyte-derived cytokine, leptin. *Microscopy Research and Technique*.

[7] Liao W.-L., Chen C.-C., Chang C.-T. (2012). Gene polymorphisms of adiponectin and leptin receptor are associated with early onset of type 2 diabetes mellitus in the Taiwanese population. *International Journal of Obesity*.

[8] Yu Z., Han S., Cao X., Zhu C., Wang X., Guo X. (2012). Genetic polymorphisms in adipokine genes and the risk of obesity: a systematic review and meta-analysis. *Obesity*.

[9] He J., Xu G. (2013). *LEP* gene variant is associated with prostate cancer but not with colorectal cancer. *Tumor Biology*.

[10] Dallal C., Garte S., Ragin C. (2013). Plasma leptin levels, LEPR Q223R polymorphism and mammographic breast density: a cross-sectional study. *The International Journal of Biological Markers*.

[11] Wang L.-Q., Shen W., Xu L. (2012). The association between polymorphisms in the leptin receptor gene and risk of breast cancer: a systematic review and pooled analysis. *Breast Cancer Research and Treatment*.

[12] Kim E.-Y., Chin H.-M., Park S.-M. (2012). Susceptibility of gastric cancer according to leptin and leptin receptor gene polymorphisms in Korea. *Journal of the Korean Surgical Society*.

[13] Li Y., Geng J., Wang Y. (2012). The role of leptin receptor gene polymorphisms in determining the susceptibility and prognosis of NSCLC in Chinese patients. *Journal of Cancer Research and Clinical Oncology*.

[14] Bufalo N. E., Leite J. L., Guilhen A. C. T. (2006). Smoking and susceptibility to thyroid cancer: an inverse association with CYP1A1 allelic variants. *Endocrine-Related Cancer*.

[15] Xu L., Morari E. C., Wei Q., Sturgis E. M., Ward L. S. (2012). Functional variations in the ATM gene and susceptibility to differentiated thyroid carcinoma. *The Journal of Clinical Endocrinology & Metabolism*.

[16] de Almeida J. F. M., Tsumura W. G., Vaisman M., Assumpção L. V. M., Ward L. S. (2012). Current recommendations for levothyroxine treatment of differentiated thyroid cancer patients are not properly implemented in a clinical practice. *Journal of Endocrinological Investigation*.

[17] Marcello M. A., Sampaio A. C., Geloneze B., Vasques A. C. J., Assumpção L. V. M., Ward L. S. (2012). Obesity and excess protein and carbohydrate consumption are risk factors for thyroid cancer. *Nutrition and Cancer*.

[18] WHO Obesity and overweight. http://www.who.int/mediacentre/factsheets/fs311/en/index.html.

[19] Cooper D. S., Doherty G. M., Haugen B. R. (2009). Revised American thyroid association management guidelines for patients with thyroid nodules and differentiated thyroid cancer. *Thyroid*.

[20] Pitoia F., Ward L., Wohllk N. (2009). Recommendations of the Latin American Thyroid Society on diagnosis and management of differentiated thyroid cancer. *Arquivos Brasileiros de Endocrinologia e Metabologia*.

[21] Camargo R., Corigliano S., Friguglietti C. (2009). Latin American thyroid society recommendations for the management of thyroid nodules. *Arquivos Brasileiros de Endocrinologia& Metabologia*.

[22] Jorge M. L. M. P., de Oliveira V. N., Resende N. M. (2011). The effects of aerobic, resistance, and combined exercise on metabolic control, inflammatory markers, adipocytokines, and muscle insulin signaling in patients with type 2 diabetes mellitus. *Metabolism: Clinical and Experimental*.

[23] Godoy-Matos A. F., Bahia L. R., Domingues R. C. (2010). Adiponectin is related to intramyocellular lipid content in non-diabetic adults. *Journal of Endocrinological Investigation*.

[24] Geloneze B., Pereira J. A., Pareja J. C. (2009). Overcoming metabolic syndrome in severe obesity: adiponectin as a marker of insulin sensitivity and HDL-cholesterol improvements after gastric bypass. *Arquivos Brasileiros de Endocrinologia e Metabologia*.

[25] Slattery M. L., Wolff R. K., Herrick J., Caan B. J., Potter J. D. (2008). Leptin and leptin receptor genotypes and colon cancer: gene-gene and gene-lifestyle interactions. *International Journal of Cancer*.

[26] García-Bermúdez M., González-Juanatey C., Rodríguez-Rodríguez L. (2011). Lack of association between LEP rs2167270 (19 G>A) polymorphism and disease susceptibility and cardiovascular disease in patients with rheumatoid arthritis. *Clinical and Experimental Rheumatology*.

[27] Jiang Y., Wilk J. B., Borecki I. (2004). Common variants in the 5′ region of the leptin gene are associated with body mass index in men from the National Heart, Lung, and Blood Institute Family Heart Study. *The American Journal of Human Genetics*.

[28] He J., Xi B., Ruiter R. (2013). Association of LEP G2548A and LEPR Q223R polymorphisms with cancer susceptibility: evidence from a meta-analysis. *PLoS ONE*.

[29] Furusawa T., Naka I., Yamauchi T. (2010). The Q223R polymorphism in LEPR is associated with obesity in Pacific Islanders. *Human Genetics*.

[30] Saukko M., Kesäniemi Y. A., Ukkola O. (2010). Leptin receptor Lys109Arg and Gln223Arg polymorphisms are associated with early atherosclerosis. *Metabolic Syndrome and Related Disorders*.

[31] Lucas A., Granada M. L., Olaizola I. (2013). Leptin and thyrotropin relationship is modulated by smoking status in euthyroid subjects. *Thyroid*.

[32] Duntas L. H., Biondi B. (2013). The interconnections between obesity, thyroid function, and autoimmunity: the multifold role of leptin. *Thyroid*.

[33] Marzullo P., Minocci A., Tagliaferri M. A. (2010). Investigations of thyroid hormones and antibodies in obesity: leptin levels are associated with thyroid autoimmunity independent of bioanthropometric, hormonal, and weight-related determinants. *Journal of Clinical Endocrinology and Metabolism*.

[34] Guzel S., Seven A., Guzel E. C., Buyuk B., Celebi A., Aydemir B. (2013). Visfatin, leptin, and TNF-*α*: interrelated adipokines in insulin-resistant clinical and subclinical hypothyroidism. *Endocrine Research*.

[35] Mammès O., Betoulle D., Aubert R., Herbeth B., Siest G., Fumeron F. (2000). Association of the G-2548A polymorphism in the 5′ region of the LEP gene with overweight. *Annals of Human Genetics*.

[36] Portoles O., Sorli J. V., Frances F. (2006). Effect of genetic variation in the leptin gene promoter and the leptin receptor gene on obesity risk in a population-based case-control study in Spain. *European Journal of Epidemiology*.

[37] Liu C., Liu L. (2011). Polymorphisms in three obesity-related genes (LEP, LEPR, and PON1) and breast cancer risk: a meta-analysis. *Tumour Biology*.

[38] Hoffsted J., Eriksson P., Mottagui-Tabar S., Arner P. (2002). A polymorphism in the leptin promoter region (-2548 G/A) influences gene expression and adipose tissue secretion of leptin. *Hormone and Metabolic Research*.

[39] Murugesan D., Arunachalam T., Ramamurthy V., Subramanian S. (2010). Association of polymorphisms in leptin receptor gene with obesity and type 2 diabetes in the local population of Coimbatore. *Indian Journal of Human Genetics*.

[40] Quinton N. D., Lee A. J., Ross R. J. M., Eastell R., Blakemore A. I. F. (2001). A single nucleotide polymorphism (SNP) in the leptin receptor is associated with BMI, fat mass and leptin levels in postmenopausal Caucasian women. *Human Genetics*.

[41] Mattevi V. S., Zembrzuski V. M., Hutz M. H. (2002). Association analysis of genes involved in the leptin-signaling pathway with obesity in Brazil. *International Journal of Obesity*.

[42] Yiannakouris N., Yannakoulia M., Melistas L., Chan J. L., Klimis-Zacas D., Mantzoros C. S. (2001). The Q223R polymorphism of the leptin receptor gene is significantly associated with obesity and predicts a small percentage of body weight and body composition variability. *Journal of Clinical Endocrinology and Metabolism*.

[43] Stefan N., Vozarova B., Del Parigi A. (2002). The Gln223Arg polymorphism of the leptin receptor in Pima Indians: influence on energy expenditure, physical activity and lipid metabolism. *International Journal of Obesity*.

[44] Chiu K. C., Chu A., Chuang L.-M., Saad M. F. (2004). Association of leptin receptor polymorphism with insulin resistance. *European Journal of Endocrinology*.

[45] Chu A., Chuang L. M., Saad M., Chiu K. (2003). Association of the Q223R polymorphism of the leptin receptor gene with insulin resistance and metabolic syndrome. *Diabetes*.

[46] Pimentel Duarte S. F., Francischetti E. A., Genelhu-Abreu V. (2006). p.Q223R leptin receptor polymorphism associated with obesity in Brazilian multiethnic subjects. *The American Journal of Human Biology*.

[47] Wauters M., Mertens I., Chagnon M. (2001). Polymorphisms in the leptin receptor gene, body composition and fat distribution in overweight and obese women. *International Journal of Obesity*.

[48] Ogawa T., Hirose H., Yamamoto Y. (2004). Relationships between serum soluble leptin receptor level and serum leptin and adiponectin levels, insulin resistance index, lipid profile, and leptin receptor gene polymorphisms in the Japanese population. *Metabolism: Clinical and Experimental*.

[49] Fairbrother U. L., Tankó L. B., Walley A. J., Christiansen C., Froguel P., Blakemore A. I. F. (2007). Leptin receptor genotype at Gln223Arg is associated with body composition, BMD, and vertebral fracture in postmenopausal Danish women. *Journal of Bone and Mineral Research*.

[50] Salopuro T., Pulkkinen L., Lindström J. (2005). Genetic variation in leptin receptor gene is associated with type 2 diabetes and body weight: the Finnish Diabetes Prevention Study. *International Journal of Obesity*.

[51] Wauters M., Mertens I., Rankinen T., Chagnon M., Bouchardt C., van Gaal L. (2001). Leptin receptor gene polymorphisms are associated with insulin in obese women with impaired glucose tolerance. *Journal of Clinical Endocrinology and Metabolism*.

[52] Park K. S., Shin H. D., Park B. L., et al (2006). Polymorphisms in the leptin receptor (LEPR)—putative association with obesity and T2DM. *Journal of Human Genetics*.

[53] Han C.-Z., Du L.-L., Jing J.-X. (2008). Associations among lipids, leptin, and leptin receptor gene Gin223Arg polymorphisms and breast cancer in China. *Biological Trace Element Research*.

[54] Okobia M. N., Bunker C. H., Garte S. J. (2008). Leptin receptor Gln223Arg polymorphism and breast cancer risk in Nigerian women: a case control study. *BMC Cancer*.

[55] He B.-S., Pan Y.-Q., Zhang Y., Xu Y.-Q., Wang S.-K. (2012). Effect of LEPR Gln223Arg polymorphism on breast cancer risk in different ethnic populations: a meta-analysis. *Molecular Biology Reports*.

[56] Lin D. W., FitzGerald L. M., Fu R. (2011). Genetic variants in the LEPR, CRY1, RNASEL, IL4, and ARVCF genes are prognostic markers of prostate cancer-specific mortality. *Cancer Epidemiology, Biomarkers & Prevention*.

[57] Li Y. L., Geng J. L., Wang Y. (2012). The role of leptin receptor gene polymorphisms in determining the susceptibility and prognosis of NSCLC in Chinese patients. *Journal of Cancer Research and Clinical Oncology*.

[58] Wazir U., Al Sarakbi W., Jiang W. G., Mokbel K. (2012). Evidence of an autocrine role for leptin and leptin receptor in human breast cancer. *Cancer Genomics and Proteomics*.

[59] Li L., Lee K. J., Choi B. C., Baek K. H. (2013). Relationship between leptin receptor and polycystic ovary syndrome. *Gene*.

[60] Friedlander Y., Li G., Fornage M. (2010). Candidate molecular pathway genes related to appetite regulatory neural network, adipocyte homeostasis and obesity: results from the CARDIA Study. *Annals of Human Genetics*.

